# Estimating the optical properties of corneal tissue from the OCT speckle

**DOI:** 10.1364/BOE.579879

**Published:** 2026-01-02

**Authors:** Maria Miażdżyk, Alejandra Consejo, D. Robert Iskander

**Affiliations:** 1 Department of Biomedical Engineering, Wroclaw University of Science and Technology, Wybrzeze Wyspianskiego 27, 50-370 Wroclaw, Poland; 2 Department of Applied Physics, University of Zaragoza, Zaragoza, Spain; 3 Aragon Institute for Engineering Research (I3A), University of Zaragoza, Zaragoza, Spain

## Abstract

Estimating the scattering coefficient *μ*_*s*_ and scattering anisotropy factor *g* of the corneal tissue is currently limited to methods utilizing double integrating spheres or spectroscopic techniques, prohibiting such corneal tissue evaluation from being performed in a clinical setting. This paper presents a new concept of statistical matching between a given corneal optical coherence tomography (OCT) scan and a set of multi-reference phantom OCT B-scans, suitably simulated using the Monte Carlo method. The statistical matching that exploits the information present in the speckle includes an ensemble of distance measures. Using a set of OCT scans from 11 porcine eyeballs, for which epithelium was removed, it is demonstrated that the proposed statistical matching approach leads to a precise estimation of the optical properties of corneal stroma. The group mean of the scattering coefficient and the scattering anisotropy factor for the porcine stroma were ${\bar{\mu }}_{s}=0.146\pm 0.020$ mm^−1^ and $\bar{g}=0.893\pm 0.021$ , respectively. These estimates match previously reported values established with other methods. The proposed approach of utilizing information present in the speckle can be readily extended to in vivo OCT imaging of human corneal tissue.

## 1. Introduction

Knowledge about the optical properties of tissue is fundamental to understand light-tissue interaction and may, in the future, be used to support medical diagnostics. Optical coherence tomography (OCT) can be used to determine these properties, particularly the scattering coefficient $\mu_{s}$ and scattering anisotropy factor g . This can be achieved using various methods that exploit the depth-dependent decay of the OCT signal [1,2].

In the near-infrared spectral range, the scattering coefficient $\mu_{s}$ of most tissues, including cornea, is much larger than the absorption coefficient $\mu_{a}$ . In this scenario, the total attenuation coefficient is approximately equal to $\mu_{s}$ . Therefore, by analyzing the slope of the OCT signal on a logarithmic scale, the scattering coefficient $\mu_{s}$ can be effectively measured but such a method requires good signal-to-noise ratio (SNR) [3]. On the other hand, the scattering anisotropy factor g can be measured from an OCT signal by fitting the depth-dependent OCT signal to a theoretical model, such as one based on the Extended Huygens-Fresnel principle [2,4]. Here, also, a good SNR is the requirement for such a derivation.

Classical approaches such as the inverse adding doubling, Kubelka–Munk, and inverse Monte Carlo methods were applied to measure the optical properties of bovine and porcine corneas, using a double integrating sphere setup [5–7] or using a spectroscopic technique [8]. Both approaches cannot be applied to in-vivo assessment of optical properties of corneal tissue. Would OCT offer a solution for this case?

The coherent nature of light in OCT leads to speckle formation. Speckle poses a significant challenge in measuring the optical properties of tissues, leading to a significant reduction in measurement accuracy. The precision of backscatter measurements in OCT signals can be significantly improved by spatially averaging the speckle [3,9]. However, the determination of tissue parameters based on raw, unaveraged OCT signals has been so far considered unfeasible using methods that relay on good SNR. Although often perceived as noise that degrades image quality, speckle patterns in OCT images can convey valuable information about the sample’s microstructure [10], and that it particularly appealing when imaging the cornea.

While statistical parameters of OCT speckle have been estimated for corneal tissue and linked to its integrity [11–13], their direct correlation with fundamental optical properties has been so far only partially resolved [14]. A significant gap exists in determining whether speckle-derived metrics can serve as reliable indicators of scattering parameters within the corneal tissue. Addressing this gap is important, as no current in-vivo methods allow direct quantification of corneal optical properties. In this study, a look-up map of simulated, via a Monte Carlo method, multi-reference phantom OCT B-scans is used to estimate via a sample-reference match the scattering coefficient $\mu_{s}$ and the scattering anisotropy factor g of an ex-vivo porcine corneal tissue from the speckle statistics. By bridging the link between corneal OCT speckle statistics and light-scattering optical properties, this work aims to advance the accuracy and clinical utility of OCT for corneal tissue characterization.

## 2. Methods

### 2.1. Porcine corneal OCT images of unknown optical properties

**Fig. 1.**
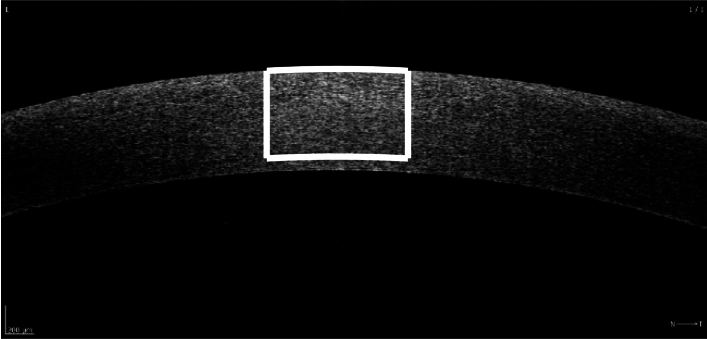
One of the porcine corneal OCT scans imaged with SOCT REVO 80 device. The images were of size 1,538 pixels horizontally and approximately 733 pixels vertically, corresponding to the estimated pixel size of 3.25 µm and 2.70 µm, respectively. White lines delineate the ROI chosen for optical parameters estimation. ROI size: 520 × 1000 µm.

A retrospective OCT image dataset recorded with SOCT REVO 80 (Optopol Technology, Poland) consisted of 11 porcine eyeballs imaged ex-vivo, after epithelial debridement. The eyeballs were obtained from a registered abattoir and came from animals from the same farm, breed, and were of approximately the same age. Intact enucleated eyeballs were immersed in a phosphate-buffered saline (PBS) solution, preserved in a portable refrigerator at $4^{\circ }$ , and prepared for experiments within 3 hours post mortem. They were further mounted on a custom tool to set and maintain an intraocular pressure of 20 mmHg in the anterior chamber. Additionally each eyeball was kept moist by continuously soaking it in the PBS solution, facilitating in this way semi-physiological conditions for OCT image acquisition. The imaging protocol consisted of acquiring three central horizontal B-scans of 5 mm width and approximately 2 mm depth, without realigning the instrument. The images were of size 1,538 pixels horizontally and approximately 733 pixels vertically, corresponding to the estimated pixel size of 3.25 µm and 2.70 µm, respectively. A detailed description of the eyeball measurement can be found in [15,16]. Epithelial debridement ensured one-layer corneal samples, to exclude epithelial layer influence on the stromal OCT speckle statistics, as the previous study has shown that epithelium significantly influences the backscattered signal from the stroma [17]. Figure 1 shows an illustrative example of the corneal OCT scan with a region of interest (ROI) outlined in white. The central ROI of 308 pixels (approximately 1000 µm) width around the corneal apex was used for deriving the speckle statistics, as it was already justified in previous study [17] for the same data set of porcine corneas. Its depth (193 pixels, approximately 520 µm) was limited by the central corneal thickness of the thinnest sample. The thickest sample in these cohort of eyeballs was 671 µm.

### 2.2. Look-up map: simulated OCT images of known tissue optical properties

**Fig. 2.**
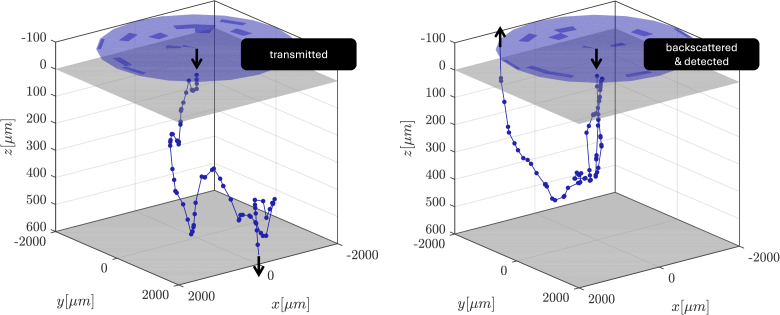
An illustrative example of the Monte Carlo simulation of the photon packet transport through the tissue of depth of 600 µm. Grey surfaces indicate tissue borders. Blue circle indicates the detector of radius 0.2 cm located 100 µm above the sample (the justification of this setup is explained in subsection 2.2). Two cases are shown: photon packet transmitted (left) and photon packet backscattered & detected (right).

To simulate photon transport through the corneal stroma, a Monte Carlo algorithm for photon transport through multilayered tissue, originally developed by Prahl [18] and Wang et al. [19], was implemented. The algorithm is based on the Mie scattering theory and uses tissue optical properties, i.e., the scattering coefficient $\mu_{s}$ , the absorption coefficient $\mu_{a}$ , the scattering anisotropy factor g and the refractive index n as input parameters. A detailed description of this Monte Carlo algorithm can be found in [19]. Here, its general workflow is summarized. The photon packet is launched at the sample surface and propagated through the tissue. Each step is tracked and managed with the mentioned set of optical properties until it exits the sample, being transmitted, backscattered, or absorbed. This operation is repeated — the subsequent photon packets are launched and traced. The number of packets should be carefully chosen and will be addressed later. Figure 2 shows illustrative examples of the photon packet paths for two cases: transmitted, as well as backscattered and detected.

Collecting the pathlength of each photon, its weight and the information if it was backscattered and hit the detector, allowed to derive the OCT signal (the A-scan) as proposed in [20]. Then, every B-scan was created with 40 A-scans, corresponding to 40 Monte Carlo runs. The simulated OCT images include the carrier frequency and the pixel values oscillate around zero, taking negative values. For this reason, they cannot be compared with experimental data. As suggested in [20], for subsequent analysis, the signal envelope was calculated in order to remove the carrier frequency. This was performed by treating the simulated OCT signal as a sum of phasors of the same frequency.

#### 2.2. OCT system setup for simulation

The look-up map was created to estimate the optical properties of corneal stroma imaged with SOCT REVO 80 device. Therefore, the OCT setup for the simulation was adapted to follow the particular technical specifications, i.e, the central wavelength was $\lambda_{0}=850$ nm and the bandwidth was Δλ=50 nm at half maximum (i.e., FWHM). The coherence length of the light source was calculated from $l_{c}=2\ln(2)\lambda_{0}^{2}/(\pi \Delta \lambda )$ and also used as an input parameter. The axial pixel size of 2.7 µm was chosen to follow the vertical resolution of the experimental OCT images described in subsection 2.1. The beam was assumed to be infinitely thin, so the lateral resolution was not considered. The resulting image was then of size 223×20 pixels, corresponding to 40 A-scans and approximately 600 µm reference depth. Every A-scan was a result of different realization of the random variable, while input parameters for simulation were constant throughout creating the whole B-scan. Therefore, the number of A-scans was justified as a trade-off between sufficient number of pixels and computational cost. Similarly as in the experimental data, the ROI for subsequent analysis was chosen. Its depth was set to 193 pixels (approximately 520 µm) to fit the thinnest experimental cornea and the width corresponded to the whole B-scan width (40 pixels).

Because the OCT signal only accounts for those photon packets that were backscattered from the sample and reached the detector after leaving the sample surface, consideration had to be given to how the detector should be positioned above the sample and its dimensions. In real OCT systems, the numerical aperture (NA) is low to maintain a larger depth of field, and therefore, the axial range. The NA can be calculated from $\text{NA}=\sqrt{2\ln2}\;\lambda_{0}/(\pi \;\delta x)$ [21], where the transversal resolution δx was experimentally determined for SOCT REVO 80 at 20 µm, and amounts to 0.0159. Setting the exact OCT detector geometry in the simulated setup would result in an unrealistically high number of photon packets used in Monte Carlo simulation. Hence, to keep that number within reason, the NA was normalized leading to a detector with radius of 0.2 cm located at 100 µm above the sample, as shown in Fig. 2.

#### 2.2. Choosing optical parameters for the look-up map

In order to examine speckle statistics and optical parameters $\mu_{s}$ and g of the tissue, a range of these parameters was considered. According to Yust et al. [6], who indirectly measured scattering parameters for bovine cornea in infrared region, the scattering coefficient $\mu_{s}$ of the corneal stroma for λ=850 nm is 0.1466, 0.1347 and 0.1348 ${\mathrm{mm}}^{-1}$ calculated with Kubelka-Munk (KM), inverse adding doubling (IAD) and inverse Monte Carlo methods, respectively. Regal et al. [8], on the other hand, applied the same methods to spectroscopy measurements of porcine eye tissue, including that of cornea. Their average estimates of the scattering coefficient of cornea for λ=810 nm, based on six eyeballs, amounted to $\mu_{s}=0.126\pm 0.049\;$ ${\mathrm{mm}}^{-1}$ and $\mu_{s}=0.106\pm 0.037$ ${\mathrm{mm}}^{-1}$ for KM and IAD, respectively. Consequently, a range of 0.1 to 0.2 ${\mathrm{mm}}^{-1}$ with step 0.01 ${\mathrm{mm}}^{-1}$ was chosen for $\mu_{s}$ (11 values). Absorption coefficient $\mu_{a}$ was set to 0, to simplify the analysis, especially since absorption in the cornea at 850 nm is weak (of order 0.01 ${\mathrm{mm}}^{-1}$ [6]). The scattering anisotropy factor g ranged from 0.85 to 0.99 with step of 0.01 (15 values). The chosen values of g were high, because the scattering coefficient in biological tissues is high in general [22–25] and additionally, in the cornea we observe strong scattering in the forward direction [6,26]. The refractive index ( n ) was chosen to simulate the experimental conditions of the real OCT measurement, so n between the detector and sample surface was set to 1 (air refractive index), n of the sample was chosen to 1.376 (corneal refractive index) and n behind the sample was set to 1.33 (aqueous humor) [27]. The final look-up map was created with 11×15 unique pairs of $\mu_{s}$ and g giving $N_{\text{SIM}}=165$ multi-reference phantom OCT images.

#### 2.2. Number of launched photon packets

**Fig. 3.**
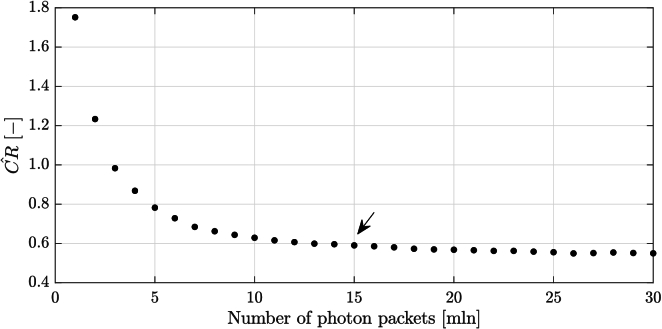
Contrast ratio of the simulated OCT B-scans and number of photon packets launched, ranging from 1 to 30 million. The plot is generated for one pair of $\mu_{s}$ and g (0.1 mm^−1^ and 0.99), the "worst" case in terms of strength of backscattered signal. The black arrow indicates the number of photon packets launched (15 million) finally chosen for creating the look-up map.

A preliminary study was conducted using the detector configuration described above to see how many photon packets are needed to generate a reliable OCT B-scan. For that, the simulation was launched for 1 to 30 million of photon packets for only one pair of $\mu_{s}$ and g . The scattering coefficient $\mu_{s}$ was set to the minimal value from the chosen range (0.1 ${\mathrm{mm}}^{-1}$ ), as the lower scattering coefficient corresponds to less scattering events, whereas g was set to the highest value (0.99) as it corresponds to small scattering angle and domination of transmission in the forward direction. This choice ensured that for the “worst” case in terms of strength of backscattered signal, there will be a sufficient number of photon packets. The contrast ratio (CR) of simulated OCT speckle was examined for the chosen ROI (see subsection 2.3) over a number of photon packets. The results have shown that for this unique pair of $\mu_{s}$ and g the mean fraction of backscattered photon packets is 0.0311% while the mean fraction of those which arrive at the detector is 0.0306%. Figure 3 shows the CR values against number of photon packets. It is noticed that CR converges approximately to the value of 0.6 and at 15 million photon packets the CR values change minimally. This point corresponds to 4595 photon packets caught at the detector, which was assumed to be the minimal number to receive a reliable B-scan. That is why for further analysis the number of launched photon packets was set to 15 million.

### 2.3. Estimation of optical properties of porcine corneas from OCT images

**Fig. 4.**
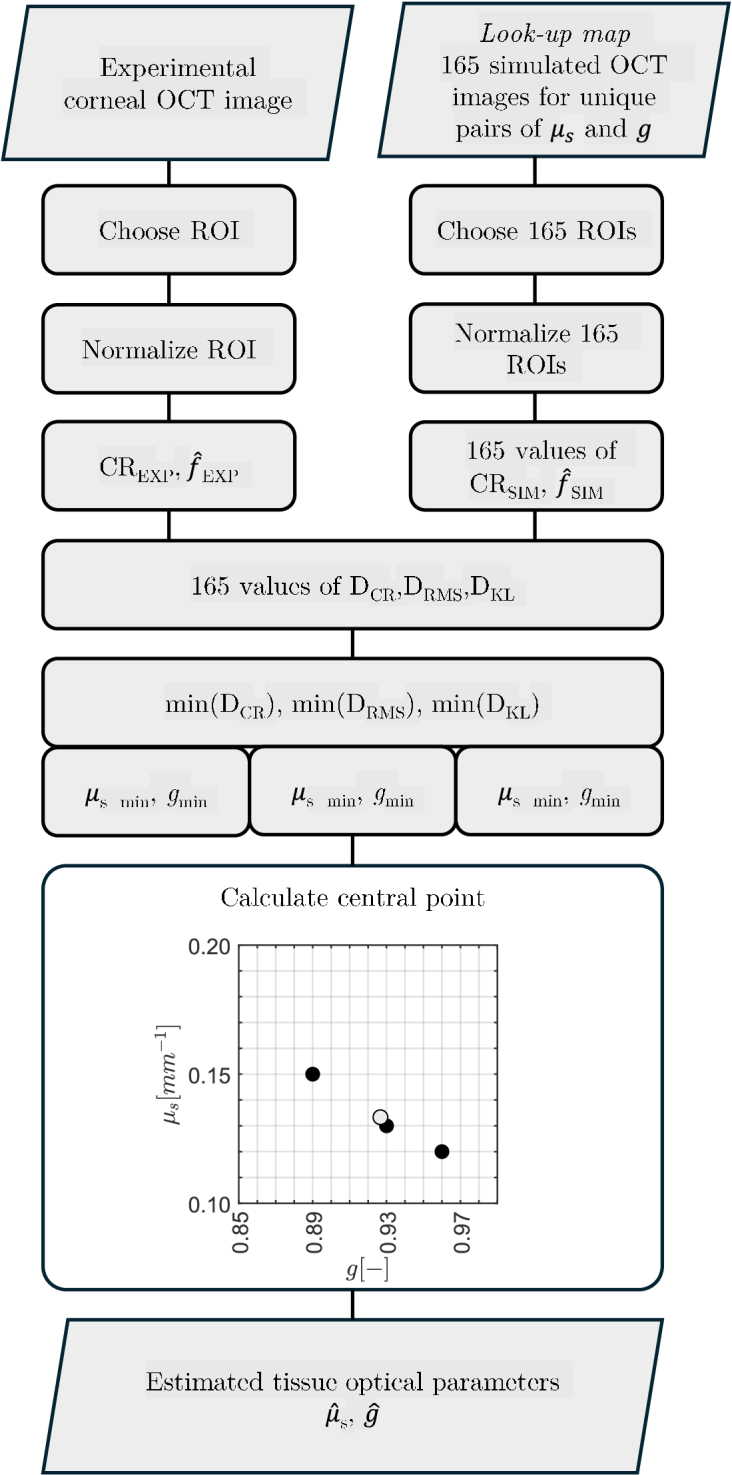
The schematic of the algorithm for estimating the scattering coefficient $\mu_{s}$ and the scattering anisotropy factor g .

The ROI data was normalized as follows: the simulated data was divided by the maximum value and then divided by the root mean square value (RMS), the experimental data was divided by 255, further divided by the RMS value and then inverse transformed using the power function, i.e., $y=10^{x}$ , where x is the normalized pixel value in the original image. To compare the simulated and experimental data, speckle statistics for every ROI were calculated including the contrast ratio (CR) and the kernel density estimator. Further, an ensemble of three distances were considered:

(1) $$D_{\text{CR}}(k)=|{\text{CR}}_{\text{EXP}}-{\text{CR}}_{k,\text{SIM}}|\text{,}$$

(2) $$D_{\text{RMS}}(k)=\sqrt{\int {\left({\hat{f}}_{\text{EXP}}(x)-{\hat{f}}_{k,\text{SIM}}(x)\right)}^{2}\;dx}\text{, and}$$

(3) $$D_{\text{KL}}(k)=\sum_{x}{\hat{f}}_{\text{EXP}}(x)\log\frac{{\hat{f}}_{\text{EXP}}(x)}{{\hat{f}}_{k,\text{SIM}}(x)}$$

as functions of the index of the simulated reference $k\in \{1,2,\ldots ,N_{\text{SIM}}\}$ , where $\hat{f}(x)$ is the kernel density estimator of the speckle statistics. $D_{\text{CR}}$ is the difference between the contrast ratios of the simulated (SIM) and experimental (EXP) ROI data, $D_{\text{RMS}}$ is the root-mean-square (RMS) error between kernel density estimators and $D_{\text{KL}}$ corresponds to the Kullback–Leibler divergence. To statistically match the ROI from a porcine stroma to one of the $N_{\text{SIM}}=165$ simulated multi-references from the look-up map, the minima of $D_{\text{CR}}(k)$ , $D_{\text{RMS}}(k)$ , and $D_{\text{KL}}(k)$ were calculated, leading to three $\mu_{s}$ and g pairs. Finally, the estimated optical properties ( ${\hat{\mu }}_{s}$ and $\hat{g}$ ) were taken as the ensemble averages across the results for the three distances. Figure 4 shows the flowchart of the described estimation method. By choosing three distances for the task of matching one is able to overcome the problem of biases present in each of the measures.

## 3. Results

**Fig. 5.**
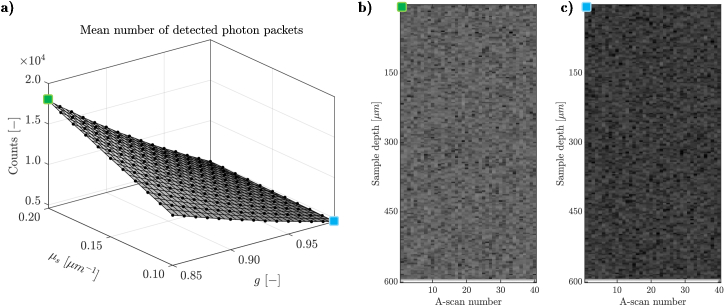
a): Mean number of photon packets (counts) caught at the detector, calculated from whole B-scan (40 A-scans). x and y axes indicate g and $\mu_{s}$ input parameters for the simulated images. The green and blue square indicate the cases shown in b) and c). b) and c): Illustrative examples of the simulated B-scan, for 2 pairs of parameters: [ $\mu_{s}=0.2$ ${\mathrm{mm}}^{-1}$ , g=0.85 ] and [ $\mu_{s}=0.1$ ${\mathrm{mm}}^{-1}$ , g=0.99 ], respectively. The images are shown after log10 transformation, as it is conventionally visualized in OCT devices.

**Fig. 6.**
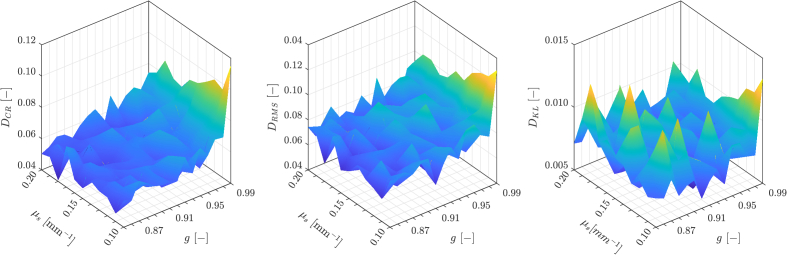
Three distance measures calculated for illustrative porcine OCT image and look-up map of 165 simulated OCT images. x and y axes indicate g and $\mu_{s}$ input parameters for the simulated images. The next step of the methodology proposed in this paper is calculating the global minimum, min(⋅) for each of the measures (see the flowchart, presented in Fig. 4).

Figure 5(a) shows the mean number of photons that hit the detector (calculated from 40 A-scans that constitute the B-scan) depending on the chosen $\mu_{s}$ and g . The observed tendencies have a straightforward physical explanation. The increase in $\mu_{s}$ follows the increase in the number of scattering particles, and if more scattering events occur, the more photons will be backscattered to the detector. The increase in g is understood as the decrease in the angle of scattering (more photons are scattered in the forward direction), so less of them will reach the detector. Exemplary log10-transformed B-scans, can be observed in Fig. 5(b) and (c). High values in the last line of pixels result from internal reflection at the tissue-medium interface (in this case it is the interface between corneal stroma and aqueous humor). Possibly, when considering absorption coefficient $\mu_{a}$ for cornea greater than 0, then the flash would be smaller. However, these pixels were excluded from further statistical analysis, as the chosen ROI depth was 193 pixels that corresponds to 521 µm. Figure 6 shows the calculated distances between the speckle statistics of B-scans from simulated multi-reference look-up map and the exemplary OCT image of the porcine cornea.

Running the Monte Carlo simulation is computationally intensive but it is an one-off task. Here, the range of $\mu_{s}$ : [0.1:0.01:0.2] ${\mathrm{mm}}^{-1}$ (11 values) and the range of g : [0.85:0.01:0.99] (15 values) resulted in 165 B-scans in the multi-reference look-up map, where every B-scan was created with unique pair of $\mu_{s}$ and g . Table 1 shows the minimum values of $D_{\text{CR}}$ , $D_{\text{RMS}}$ and $D_{\text{KL}}$ achieved for each corneal OCT acquisition of each eyeball and corresponding to them pair of $\mu_{s}$ and g read from the multi-reference look-up map. The last two columns of Table 1 show the final (mean) estimates of ${\hat{\mu }}_{s}$ and $\hat{g}$ acquired from the three considered distance measures. The group means of these parameters for the 11 eyeballs amounted to ${\bar{\mu }}_{s}=0.146\pm 0.020$ ${\mathrm{mm}}^{-1}$ and $\bar{g}=0.893\pm 0.021$ .

**Table 1.** The results of $\mu_{s}$ and g estimated using three distance measures between the given sample speckle statistics and the ones from the simulated look-up map.

		$D_{\text{CR}}$			$D_{\text{RMS}}$			$D_{\text{KL}}$			final estimates	
eyeball	meas.	min(⋅)	$\mu_{s}$	g	min(⋅)	$\mu_{s}$	g	min(⋅)	$\mu_{s}$	g	${\hat{\mu }}_{s}$	$\hat{g}$
1	1	0.0200	0.18	0.85	0.0403	0.10	0.91	0.0027	0.10	0.91	0.127	0.890
	2	0.0167	0.18	0.85	0.0380	0.14	0.93	0.0023	0.10	0.91	0.140	0.897
	3	0.0151	0.18	0.85	0.0379	0.14	0.93	0.0023	0.10	0.91	0.140	0.897
2	1	0.0437	0.18	0.85	0.0609	0.18	0.85	0.0078	0.19	0.89	0.183	0.863
	2	0.0407	0.18	0.85	0.0626	0.18	0.85	0.0082	0.19	0.89	0.183	0.863
	3	0.0439	0.18	0.85	0.0605	0.18	0.85	0.0077	0.19	0.89	0.183	0.863
3	1	0.0076	0.18	0.85	0.0314	0.10	0.91	0.0022	0.12	0.92	0.133	0.893
	2	0.0194	0.18	0.85	0.0346	0.17	0.92	0.0024	0.12	0.92	0.157	0.897
	3	0.0177	0.18	0.85	0.0360	0.17	0.92	0.0025	0.12	0.92	0.157	0.897
4	1	0.0264	0.18	0.85	0.0649	0.10	0.91	0.0080	0.10	0.91	0.127	0.890
	2	0.0275	0.18	0.85	0.0668	0.10	0.91	0.0081	0.10	0.91	0.127	0.890
	3	0.0231	0.18	0.85	0.0633	0.10	0.91	0.0071	0.10	0.91	0.127	0.890
5	1	0.0005	0.10	0.96	0.0349	0.10	0.91	0.0044	0.14	0.97	0.113	0.947
	2	0.0000	0.12	0.95	0.0353	0.10	0.91	0.0043	0.14	0.97	0.120	0.943
	3	0.0001	0.12	0.97	0.0322	0.10	0.91	0.0040	0.14	0.97	0.120	0.950
6	1	0.0431	0.18	0.85	0.0488	0.18	0.85	0.0040	0.17	0.92	0.177	0.873
	2	0.0349	0.18	0.85	0.0434	0.18	0.85	0.0032	0.17	0.92	0.177	0.873
	3	0.0386	0.18	0.85	0.0461	0.18	0.85	0.0037	0.17	0.92	0.177	0.873
7	1	0.0256	0.18	0.85	0.0419	0.14	0.93	0.0030	0.12	0.92	0.147	0.900
	2	0.0192	0.18	0.85	0.0398	0.17	0.92	0.0032	0.12	0.92	0.157	0.897
	3	0.0189	0.18	0.85	0.0377	0.10	0.91	0.0027	0.12	0.92	0.133	0.893
8	1	0.0455	0.18	0.85	0.0577	0.18	0.85	0.0054	0.12	0.92	0.160	0.873
	2	0.0467	0.18	0.85	0.0565	0.18	0.85	0.0051	0.12	0.92	0.160	0.873
	3	0.0453	0.18	0.85	0.0545	0.18	0.85	0.0051	0.12	0.92	0.160	0.873
9	1	0.0409	0.18	0.85	0.0572	0.14	0.93	0.0054	0.12	0.92	0.147	0.900
	2	0.0413	0.18	0.85	0.0577	0.14	0.93	0.0054	0.12	0.92	0.147	0.900
	3	0.0352	0.18	0.85	0.0528	0.14	0.93	0.0047	0.12	0.92	0.147	0.900
10	1	0.0140	0.18	0.85	0.0405	0.10	0.91	0.0034	0.12	0.92	0.133	0.893
	2	0.0147	0.18	0.85	0.0416	0.10	0.91	0.0032	0.12	0.92	0.133	0.893
	3	0.0156	0.18	0.85	0.0438	0.10	0.91	0.0034	0.12	0.92	0.133	0.893
11	1	0.0034	0.18	0.85	0.0299	0.10	0.91	0.0021	0.12	0.92	0.133	0.893
	2	0.0021	0.18	0.85	0.0294	0.10	0.91	0.0023	0.12	0.92	0.133	0.893
	3	0.0019	0.18	0.85	0.0254	0.10	0.91	0.0018	0.12	0.92	0.143	0.893

## 4. Discussion

Estimating the optical characteristic of the corneal tissue in-vivo is challenging. OCT technology appears to be the most suitable technique for this task but estimating such characteristics from the OCT signal requires good SNR and sufficiently strong decay of the OCT signal amplitude into the tissue depth. That is why, till now, the OCT-based techniques for determining tissue parameters were not applied to cornea. The proposed statistical approach of matching the sample speckle statics of tissue to those of the look-up map of simulated multi-reference phantom OCT B-scans overcomes these barriers because it requires neither of the conditions mentioned above. Moreover, it allows such matching using a single non-averaged corneal OCT scan. Although the proposed technique was applied here to ex-vivo porcine eyes, its extension to in-vivo measurements is feasible. In principle, this can be achieved by simulating multi-reference surfaces (such as proposed here with $D_{\text{CR}}$ , $D_{\text{RMS}}$ and $D_{\text{KL}}$ ), taking into account the two main layers of the cornea and the influence of the epithelium on the stromal backscatter statistics [17].

The estimated group mean values of the scattering coefficient and scattering anisotropy factor correspond well to those reported previously for porcine corneas [8] and bovine corneas [6]. The repeatability of the estimates using the proposed statistical matching method is noteworthy. It can be concluded that, although the speckle is a random variable, its statistics depend on the specific optical parameters of the tissue and its microstructure and carry physical useful information. The high repeatability of the estimated optical parameters confirms the high power of the developed estimation method. The obtained high repeatability was expected, as the porcine eyes sampled for the experiment came from the same registered abattoir and were of the same breed and age.

Although the direct clinical utility and translational significance of the study remain to be determined, it is important to remember that it is more convenient to model corneal backscatter using well-defined physical parameters, as in this study, than using a set of statistical models fitted to the data (e.g., Gamma, Burr, and others), whose parameters are difficult to interpret [11–14,16,28].

The study has several limitations. The Monte Carlo simulation algorithm employed here makes several simplifications. The light source beam is assumed to be infinitely thin, and the next step is to apply a Gaussian beam with a defined radius. This would introduce lateral resolution into the simulated OCT images. Furthermore, including a non-zero absorption coefficient could alter the intensity profile of the A-scan and affect the internal reflection on the last two lines of the simulated image. Corneal curvature was also omitted at this stage, and the digital sample was assumed flat, which certainly affects the number of photons reaching the detector and their path. These simplifications can be considered a limitation, but they also allow for the separation of the number of factors influencing the signal. This allows for future refinement of the algorithm, ensuring strict control of their impact on speckle statistics.

Also, one may debate whether the Mie scattering theory, which is involved in the Monte Carlo algorithm is adequate for simulating the photon transport through corneal tissue. According to Meek et al. [26], the corneal stroma consists of collagen fibers, keratocytes, which constitute up to 17% of the substance and of extracellular matrix. Due to this composition, as in most of the tissues, two types of scattering are present in the photon transport: the Mie and Rayleigh scattering [26,29]. Since the Rayleigh scattering can be considered as a simplification of the Mie theory, the latter appears to be still applicable [22,30]. One could also consider, whether the simulation is suitable for generating OCT speckle pattern. The photon packet backscattered to the detector is specified with total path traveled, which is equivalent here to the path of the sample beam in the interferometer. The equation proposed by Kirillin et al. [20], utilized for OCT signal calculation, incorporates the difference in the paths between the reference and sample beams, directly transformable via expression 2π/λ to the difference in phases. Hence, the speckle pattern is included by multiplication of the signal by the cosine of the phase difference. This key feature of speckle modeling approach is described in detail in [20].

Further, when matching the sample statistics to those of the multi-reference look-up map three measures were considered. Their individual performances and potential biases were not examined because the available sample of data is relatively small. It is important to note that they do not exhaust the possibility of a better or even optimal (in some sense) measure that could support or replace them. One may consider measures based on the speckle distribution function or speckle characteristic function as they were considered in [13]. A commonly used Monte Carlo algorithm was adapted here for ophthalmic OCT and corneal imaging, with validated OCT system parameters and optical tissue parameters based on the literature. The results indicate that this algorithm adequately simulate OCT synthetic data, which can serve as digital phantoms, with close to reality speckle statistics.

The method for generating OCT phantoms adopted in this study does not exhaust the possibilities of digital simulation of OCT speckle patterns [31,32]. Further comparative studies are needed to determine whether any of them can be considered optimal for generating corneal phantoms.

In summary, the proposed approach of matching the speckle statistics of a sample to those of a specific digital phantom with known optical light transport characteristics opens the possibility of using OCT technology in the assessment of biological tissues, which otherwise could not be performed in an in vivo clinical setting.

## Data Availability

Data underlying the results presented in this paper are not publicly available at this time but may be obtained from the authors upon reasonable request.

## References

[1] Choudhury N. Jacques S. L., “Extracting scattering coefficient and anisotropy factor of tissue using optical coherence tomography,” in *Optical Interactions with Tissue and Cells XXIII* , vol. 8221 (SPIE, 2012), pp. 144–148.

[2] Kodach V. Faber D. Van Marle J. et al., “Determination of the scattering anisotropy with optical coherence tomography,” Opt. Express 19(7), 6131–6140 (2011). 10.1364/OE.19.006131 21451637

[3] Vermeer K. A. Mo J. Weda J. J. et al., “Depth-resolved model-based reconstruction of attenuation coefficients in optical coherence tomography,” Biomed. Opt. Express 5(1), 322–337 (2014). 10.1364/BOE.5.000322 PMC3891343 24466497

[4] Thrane L. Frosz M. H. Levitz D. et al., “Extraction of tissue optical properties from optical coherence tomography images for diagnostic purposes,” in *Saratov Fall Meeting 2004: Optical Technologies in Biophysics and Medicine VI* , vol. 5771 (SPIE, 2005), pp. 139–150.

[5] Sardar D. K. Yust B. G. Barrera F. J. et al., “Optical absorption and scattering of bovine cornea, lens and retina in the visible region,” Lasers Med. Sci. 24(6), 839–847 (2009). 10.1007/s10103-009-0677-0 19495828 PMC2923491

[6] Yust B. G. Mimun L. C. Sardar D. K., “Optical absorption and scattering of bovine cornea, lens, and retina in the near-infrared region,” Lasers Med. Sci. 27(2), 413–422 (2012). 10.1007/s10103-011-0927-9 21556925 PMC4836624

[7] Yuzhakov A. V. Sviridov A. P. Baum O. I. et al., “Optical characteristics of the cornea and sclera and their alterations under the effect of nondestructive 1.56-*μ*m laser radiation,” J. Biomed. Opt. 18(5), 058003 (2013). 10.1117/1.JBO.18.5.058003 23722454

[8] Regal S. O’Connor D. Brige P. et al., “Determination of optical parameters of the porcine eye and development of a simulated model,” J. Biophotonics 12(11), e201800398 (2019). 10.1002/jbio.201800398 31251453

[9] Kholodnykh A. I. Petrova I. Y. Larin K. V. et al., “Precision of measurement of tissue optical properties with optical coherence tomography,” Appl. Opt. 42(16), 3027–3037 (2003). 10.1364/AO.42.003027 12790454

[10] Schmitt J. M. Xiang S. Yung K. M., “Speckle in optical coherence tomography,” J. Biomed. Opt. 4(1), 95–105 (1999). 10.1117/1.429925 23015175

[11] Jesus D. A. Iskander D. R., “Assessment of corneal properties based on statistical modeling of OCT speckle,” Biomed. Opt. Express 8(1), 162–176 (2017). 10.1364/BOE.8.000162 28101409 PMC5231290

[12] Iskander D. R. Kostyszak M. A. Jesus D. A. et al., “Assessing corneal speckle in optical coherence tomography: a new look at glaucomatous eyes,” Optom. Vis. Sci. 97(2), 62–67 (2020). 10.1097/OPX.0000000000001476 32011576

[13] Niemczyk M. Iskander D. R., “Statistical analysis of corneal OCT speckle: a non-parametric approach,” Biomed. Opt. Express 12(10), 6407–6421 (2021). 10.1364/BOE.437937 34745745 PMC8547992

[14] Ge G. R. Rolland J. P. Parker K. J., “Speckle statistics of biological tissues in optical coherence tomography,” Biomed. Opt. Express 12(7), 4179 (2021). 10.1364/BOE.422765 34457407 PMC8367221

[15] Niemczyk M. Danielewska M. E. Kostyszak M. A. et al., “The effect of intraocular pressure elevation and related ocular biometry changes on corneal OCT speckle distribution in porcine eyes,” PLoS One 16(3), e0249213 (2021). 10.1371/journal.pone.0249213 33770135 PMC7997020

[16] Danielewska M. E. Kostyszak M. A. Sareło P. et al., “Indirectly assessing changes in corneal properties with OCT speckle after crosslinking in porcine eyes,” Exp. Eye Res. 219, 109051 (2022). 10.1016/j.exer.2022.109051 35367416

[17] Miazdzyk M. Consejo A. Iskander D. R., “OCT based corneal densitometry: the confounding effect of epithelial speckle,” Biomed. Opt. Express 14(8), 3871 (2023). 10.1364/BOE.489054 37799674 PMC10549732

[18] Prahl S. A., “A Monte Carlo model of light propagation in tissue,” in *Dosimetry of Laser Radiation in Medicine and Biology* , vol. 10305 Mueller G. J. Sliney D. H. Potter R. F., eds., International Society for Optics and Photonics (SPIE, 1989), p. 1030509.

[19] Wang L. Jacques S. L. Zheng L., “MCML—Monte Carlo modeling of light transport in multi-layered tissues,” Comput. Methods Programs Biomed. 47(2), 131–146 (1995). 10.1016/0169-2607(95)01640-F 7587160

[20] Kirillin M. Y. Farhat G. Sergeeva E. A. et al., “Speckle statistics in OCT images: Monte Carlo simulations and experimental studies,” Opt. Soc. Am. 39(12), 3472–3475 (2014). 10.1364/OL.39.003472 24978514

[21] Aumann S. Donner S. Fischer J. et al., *Optical Coherence Tomography (OCT): Principle and Technical Realization* (Springer International Publishing, 2019), pp. 59–85. 32091846

[22] Jacques S. L., “Optical properties of biological tissues: a review,” Phys. Med. Biol. 58(14), 5007–5008 (2013). 10.1088/0031-9155/58/14/5007 23666068

[23] Welch A. J. van Gemert M. J. C. Star W. M. et al., *Overview of Tissue Optics* (Springer US, 1995), chap. 2, p. 38.

[24] Hall G. Jacques S. L. Eliceiri K. W. et al., “Goniometric measurements of thick tissue using monte carlo simulations to obtain the single scattering anisotropy coefficient,” Biomed. Opt. Express 3(11), 2707–2719 (2012). 10.1364/BOE.3.002707 23162710 PMC3493220

[25] Tycho A. Jørgensen T. M. Yura H. T. et al., “Derivation of a monte carlo method for modeling heterodyne detection in optical coherence tomography systems,” Appl. Opt. 41(31), 6676–6691 (2002). 10.1364/AO.41.006676 12412659

[26] Meek K. M. Knupp C. Lewis P. N. et al., “Structural control of corneal transparency, refractive power and dynamics,” Eye (2024). 10.1038/s41433-024-02969-7 PMC11885422 38396030

[27] Patel S. Tutchenko L., “The refractive index of the human cornea: A review,” Contact Lens and Anterior Eye 42(5), 575–580 (2019). 10.1016/j.clae.2019.04.018 31064697

[28] Danielewska M. E. Antończyk A. De Jesus D. A. et al., “Corneal optical coherence tomography speckle in crosslinked and untreated rabbit eyes in response to elevated intraocular pressure,” Trans. Vis. Sci. Tech. 10(5), 2 (2021). 10.1167/tvst.10.5.2 PMC8088219 34003977

[29] Jacques S. L., “Fractal nature of light scattering in tissues,” J. Innovative Opt. Health Sci. 04(01), 1–7 (2011). 10.1142/S1793545811001289

[30] Bohren C. F. Huffman D. R., *Particles Small Compared with the Wavelength* (John Wiley & Sons, Ltd, 1998), chap. 5, pp. 130–157.

[31] Xiong G. Xue P. Wu J. et al., “Particle-fixed Monte Carlo model for optical coherence tomography,” Opt. Express 13(6), 2182–2195 (2005). 10.1364/OPEX.13.002182 19495106

[32] Mao J. Ling Y. Xue P. et al., “Monte Carlo-based full-wavelength simulator of Fourier-domain optical coherence tomography,” Biomed. Opt. Express 13(12), 6317–6334 (2022). 10.1364/BOE.475428 36589559 PMC9774871

